# Single‐ versus multi‐model in the deep learning prediction of monitor units per control point for automated treatment planning in prostate cancer

**DOI:** 10.1002/acm2.70229

**Published:** 2025-08-29

**Authors:** Mathieu Gaudreault, Lachlan McIntosh, Katrina Woodford, Jason Li, Susan Harden, Sandro Porceddu, Vanessa Panettieri, Nicholas Hardcastle

**Affiliations:** ^1^ Peter MacCallum Cancer Centre Melbourne Victoria Australia; ^2^ Sir Peter MacCallum Department of Oncology The University of Melbourne Melbourne Victoria Australia; ^3^ Central Clinical School Monash University Melbourne Victoria Australia; ^4^ Department of Medical Imaging and Radiation Sciences Monash University Clayton Victoria Australia; ^5^ Centre for Medical Radiation Physics University of Wollongong Wollongong New South Wales Australia

**Keywords:** Artificial intelligence, Deep learning, Monitor units per control point, Prostate cancer

## Abstract

**Background:**

In contemporary radiation therapy, the radiation is modulated to conform the prescription dose to the tumor and spare organs at risk. The modulation results from a complex mathematical calculation that requires several iterations to reach a satisfactory solution, delaying treatment. The monitor units (MU) per control point (CP) control the dose magnitude and may be predicted by deep learning, a type of artificial intelligence (AI).

**Purpose:**

To introduce deep learning methods to predict the MU per CP in the context of AI volumetric modulated arc therapy (VMAT) treatment plan prediction for prostate cancer.

**Methods:**

Patients treated for prostate cancer with 60 Gy in 20 fractions between 01/2019 and 06/2024 were considered for inclusion. Two approaches were considered: a *single‐model* approach, trained on all samples, and a *multi‐model* approach, with separate models trained by CP. The inputs were either the three‐dimensional (3D) dose per CP (3D single‐model / 3D multi‐model) or the two‐dimensional (2D) average dose intensity projection per CP (2D single‐model / 2D multi‐model). The outputs were the MU per CP, which were converted to meterset weight per CP and MU per beam to create an AI‐Radiation Therapy Plan (AI‐RTPlan) with other clinical parameters retained. Clinical goals achieved with the calculated dose distribution from the AI‐RTPlan and clinical plan were compared.

**Results:**

The cohort was split into 220/40/42 homogeneous plans in the training/validation/testing dataset. Relative to the clinical case, the errors in meterset weight per CP were mean ± SD = −0.4 ± 3.8%/−0.2 ± 4.8% in 2D/3D single‐model and 0.01 ± 3.9%/‐0.1 ± 5.0% in 2D/3D multi‐model. The errors in MU per beam were −0.9 ± 5.5%/−1.2 ± 4.5% in 2D/3D single‐model and 0.4 ± 4.8%/0.5 ± 5.2% in 2D/3D multi‐model. In 2D/3D models, at least 93%/81% of patients had the same or more clinical goals achieved with AI‐RTPlans.

**Conclusions:**

Accurate prediction of MU per CP is feasible in VMAT prostate cancer treatment.

## 1 INTRODUCTION

In radiation therapy, the therapeutic goals of delivering the prescription dose to the tumor while minimizing the dose to adjacent organs at risk (OAR) are achieved through modulation of the radiation dose.^1^ The modulation is produced by varying machine parameters such as dose rate, gantry speed, and field shape.^2^ The machine parameter values result from a mathematical calculation optimizing the desired dose distribution based on objectives set on the target and OAR. The planned machine parameter values are stored in radiation therapy plan DICOM files (RTPlan) at specific control points (CP) for treatment. The process of generating a radiation therapy treatment plan involves iterations of dose optimization and calculation, which could take from hours to days before achieving a satisfactory solution.^3^ This increases the time from simulation to treatment and requires large human resources in radiation therapy centers. Thus, any effort to reduce the time to produce a treatment plan is desirable.

A novel approach to treatment planning is treatment plan prediction by deep learning.^4^ Deep learning is a type of artificial intelligence (AI) where a model is trained to perform predictions from labeled clinical datasets.^5^ In this concept, a three‐dimensional dose distribution, predicted from a computed tomography (CT) image and organ segmentations, serves as input to an AI architecture to predict the parameters needed for the machine to deliver the radiation dose. Treatment plan prediction has the potential to increase treatment quality with improved consistent planning and reduce the time between simulation and treatment.^6^ Deep learning prediction of machine parameters in volumetric modulated arc therapy (VMAT) treatment is in its early stage.^7^, ^8^ In this context, the goal is to predict machine parameters controlling the dose magnitude and shape. The former may be obtained from the prediction of the monitor units per control point, from which the meterset weight per control point and monitor units per beam may be derived.

Few studies investigated deep learning predictions of machine parameters for VMAT treatment. A pioneering approach focused on predicting only the beam shape for one‐arc VMAT in prostate cancer.^9^ Furthermore, two innovative studies predicted both the dose magnitude and shape, one from the dose distribution in prostate cancer,^7^ and the other from the planning CT image in breast cancer.^8^ The feasibility of using deep learning methods to predict machine parameters was demonstrated in these studies.

For this workflow to be valid and integrated into the clinical routine, the accuracy of each prediction (organ segmentation prediction, dose distribution prediction, multileaf collimator (MLC) leaf positions prediction, and monitor units per control point prediction) must be optimal, and therefore any improvements are beneficial. With the global aim to generate a framework able to predict deliverable radiation treatment plans with minimal input, we propose to simplify the problem into building blocks and address the sole prediction of dose magnitude in modulated arc treatment. We hypothesize that a deep learning approach can predict the meterset weight per control point and monitor units per beam with sufficient accuracy for plans to be clinically acceptable.

In this study, we aim to introduce a machine‐learning workflow predicting the monitor units per control point in curable prostate cancer. In deep learning, high variability may improve the model performance on unseen data and prevent overfitting whilst low variability in the training dataset may help the model to learn patterns and improve convergence.^10^ Therefore, we further aim to compare two methods to perform predictions, a single‐model approach that predicts monitor units for any control point (high variability), and a multi‐model approach, in which each model is specifically trained per control point (low variability).

## 2 MATERIALS AND METHODS

### 2.1 Treatment plans

Consecutive patients treated with curative intent for prostate cancer without lymph node involvement between 01/2019 and 06/2024 in a single institution were considered for inclusion. Ethics approval was provided by the Human Research Ethics Committee of the Peter MacCallum Cancer Centre (HREC/101771/PMCC). Patients were selected to comprise a homogeneous set of treatment plans, which was split into training/validation/testing datasets. The validation dataset was used during training to assess the model performance, whilst the testing dataset consisted of unseen data during training and was used to make predictions. The clinical tumor volume was segmented as the whole prostate augmented by 1 cm of the proximal vesicles by radiation oncologists. The prescription dose was 60 Gy in 20 daily fractions, 5 days per week over 4 weeks. All VMAT plans consisted of two counter‐rotating arcs of 360° with control point at each 2° (180 control points per arc). The collimator was rotated by ± 5° or ± 15°_._ The MLC had 60 leaf pairs, where inner leaves had 5 mm width and outer leaves had 10 mm width. Dose optimization and dose calculation were performed with the Eclipse treatment planning system (TPS) (v16.1, Varian Medical Systems, Palo Alto, USA) using the Photon Optimizer algorithm and the AcurosXB algorithm, reporting dose to medium, respectively.

### 2.2 Workflow

**FIGURE 1:**
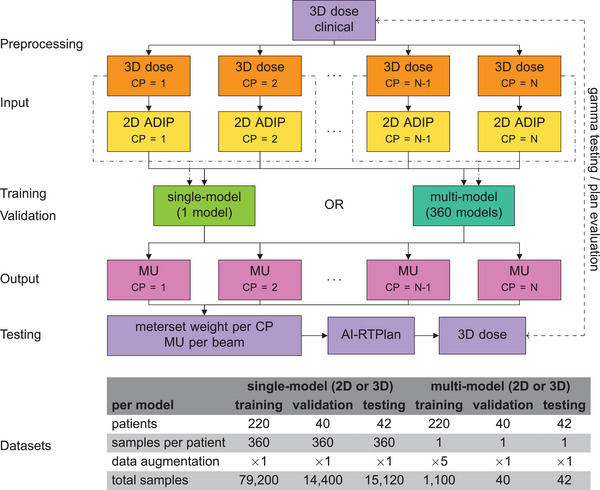
Workflow used in this study. Three‐dimensional (3D) dose per control point (CP) were generated and processed as two‐dimensional (2D) average dose intensity projection (ADIP) images. The 3D doses or 2D ADIPs per CP were the inputs of the single‐model and multi‐model approaches. The outputs were the predicted monitor units (MU) per CP, which were converted to meterset weight per CP and MU per beam into an Artificial Intelligence‐Radiation Therapy Plan (AI‐RTPlan). The AI‐RTPlan was automatically imported into the treatment planning system for dose calculation. The resulting three‐dimensional dose distribution was compared with the clinical dose distribution with gamma testing and dose evaluation. The number of patients, samples per patient, level of data augmentation, and total samples per model used at the training, validation, and testing stages are shown.

The workflow of this study is shown in Figure 1. The network architecture assumed that the three‐dimensional dose distribution per control point was provided for each control point. To obtain these dose distributions, the clinical dose distribution was split by using the BeamSplitter, a validated algorithm providing the dose per control point introduced by our research group.^11^ The dose voxel size was 2.5 mm × 2.5 mm × 2.0 mm. The meterset weights per control point were extracted from the clinical treatment plan. The monitor units per control point were determined with a previously validated method implemented by our research group.^12^

### 2.3 Deep learning approaches

Two different approaches to predict the monitor units using deep convolutional neural networks were tested and compared. In the first approach, a single model was trained by using all dose distributions and their associated monitor units in the training dataset (the *single‐model* approach). In the second approach, multiple models were trained specialized on one control point and used only dose distributions and their associated monitor units of this control point (the *multi‐model* approach). Each approach was further tested with two dimensionalities as input: (1) the three‐dimensional dose distribution per control point (3D single‐model/3D multi‐model) and (2) the two‐dimensional average dose intensity projection (ADIP) along the beam‐eye view direction of the dose distribution per control point (2D single‐model/2D multi‐model). Therefore, four scenarios to predict monitor units per control point were tested. Given the proximity to clinical plans, another approach was tested to investigate the necessity of an AI workflow. This approach used the mean of the monitor units over all patients for each control point (the average approach) to generate RTPlans.

### 2.4 Preprocessing

To center the input, a structure was generated from the clinical dose distribution by taking all voxels with dose ≥ 70% of the maximum dose in the clinical plan. The dose distribution per control point was cropped to produce an image of 64 × 64 × 64 voxels centered at the centroid of the above structure. To generate the ADIP, the dose distribution was first rotated by the inverse of the gantry angle before cropping. The resulting 3D image was then averaged along the beam‐eye view direction.

### 2.5 Architecture

**FIGURE 2:**
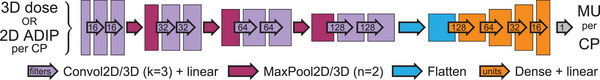
Model's architecture used in the single‐model and multi‐model approaches. The inputs were either the three‐dimensional dose distribution (3D dose) or the two‐dimensional average dose intensity projection (2D ADIP) per control point (CP) whilst the outputs were the monitor units (MU) per CP.

The deep convolutional neural network architecture used is shown in Figure 2. It consisted of one encoder followed by densely connected layers. Four downsampling steps made of two consecutive 2D or 3D convolutions with *k* = 3 kernel size and linear activation followed by a maximum pooling layer with *n* = 2 window size, were used. The filter depth of the first layer was 16 and was increased by a factor of 2 at each step. The output of the downsampling steps was flattened and passed to four consecutive densely connected layers with linear activation and a decreasing number of neuron units. The total parameter count was 566,385 (2.16 MB) in 2D models and 1,938,321 (7.39 MB) in 3D models. The models were trained with NVIDIA Tesla V100 graphics processing units (GPU) with 32 GB of video random access memory (VRAM). Inference was performed with one NVIDIA A100 GPU equipped with 80 GB of VRAM. The Adaptive Moment Estimation (Adam) optimizer was used^13^ with a learning rate of 0.001 and the Adaptive Moment Estimation with Maximum Second Moment (AMSGrad) option.^14^ The AMSGrad option stabilizes optimization in the training process. The learning rate was reduced by a factor of 0.2 after every five epochs without improvement in the loss function with a cooldown period of five epochs up to a minimum learning rate of 10^−6^. The loss function was driven by the mean absolute percentage (MAPE) difference between the true and predicted monitor units. Training was stopped automatically after 10 epochs with no improvements in the loss function. The best model achieved during training was saved. Implementation was done in Python with the Tensorflow module (version 2.15).

### 2.6 Data augmentation

Due to the relatively small sample size in the multi‐model approach, training data were augmented. Since only the dose magnitude was of interest, the images and monitor units were rescaled four times by a random number drawn from a normal distribution with mean = 100 with variance = 10. The parameters were chosen to ensure no negative random numbers. This condition was further monitored during training. The original image and monitor units were multiplied by a factor of 100, therefore, data augmentation increased the training sample size by a factor of five.

### 2.7 *K*‐fold cross‐validation

The *k*‐fold cross‐validation strategy was used to increase performance,^15^ which consists of repeated training (named fold) with overlapping samples in the training and validation datasets between models.^16^ Each fold resulted in an individual model to be applied on the testing dataset. Predictions of each fold were averaged to generate a single output. To balance inference times with model's accuracy, we used a five‐fold cross‐validation for each of the four scenarios studied. The folds were constructed on a per‐patient basis to avoid data leakage. Class imbalance was assessed by monitoring differences in training and validation MAPE between folds. In multi‐models, the average MAPE over all control points per fold was considered for this assessment. The classes were judged balanced across folds if the difference in MAPE was lower than 5%.

### 2.8 Postprocessing

The outputs of the model were the monitor units for each control point. The monitor units per beam were obtained by summing their corresponding monitor units per control point. The meterset weights per control point associated with a beam were derived by normalizing all monitor units per control point with the monitor units for the beam. Predicted and clinical meterset weights per control point and monitor units per beam were compared to the clinical value by calculating the mean and standard deviation of the relative error. Furthermore, assuming a linear relationship, correlations between predicted and clinical monitor units and meterset weights were calculated with the Pearson correlation coefficient (r) and its associated *p*‐value.

### 2.9 Dose evaluation

To evaluate dose, clinical RTPlans of the test dataset were overwritten to create an AI‐RTPlan with predicted meterset weight per control point and monitor units per beam. The resulting AI‐RTPlans were imported into the TPS for dose calculation. The importation was fully automated by using the Eclipse DB daemon and the DICOM toolkit software (DCMTK, v3.6.5). Per our clinical guidelines, the resulting dose distribution was normalized when appropriate to maximize the number of clinical goals achieved. The normalized dose distribution was compared with the clinical dose distribution with gamma testing and passing rate.^17^ The calculation was performed by using Python with the pymedphys module (v0.39.3) with 3%/3 mm criteria and a dose threshold of 10%.^18^

Target and organs at risk dose‐volume metrics and the number of clinical goals achieved from the AI‐RTPlan were compared with those of the clinical plan. The dose‐volume metrics evaluated are shown in Table 1. The absolute difference between dose‐volume metrics per patient in AI‐RTPlans and clinical plans (ΔD in percentage of prescribed dose or Δ*V* in percentage of the total volume) was further calculated. Plan quality for a given patient was evaluated with the number of clinical goals achieved and the absolute difference between dose‐volume metrics.

**TABLE 1:** Clinical goals used in this study.

Site	Objective		Dose limit
PTV	D99%	>	57 Gy
Bladder	V30Gy	<	40%
	V40Gy	<	25%
Femoral head left	V60Gy	<	1%
	V45Gy	<	5%
Femoral head right	V60Gy	<	1%
	V45Gy	<	5%
Rectum	V20Gy	<	55%
	V30Gy	<	40%
	V35Gy	<	30%
	V50Gy	<	20%
	V57Gy	<	10%
	V60Gy	<	1%
Bowel	D2%	<	45 Gy

*Note*: Dose limits are expressed in Gy or as a percentage of the prescribed dose (%).

Abbreviations: D, Dose; PTV, planning target volume; V, volume.

## 3 RESULTS

### 3.1 Datasets

**FIGURE 3:**
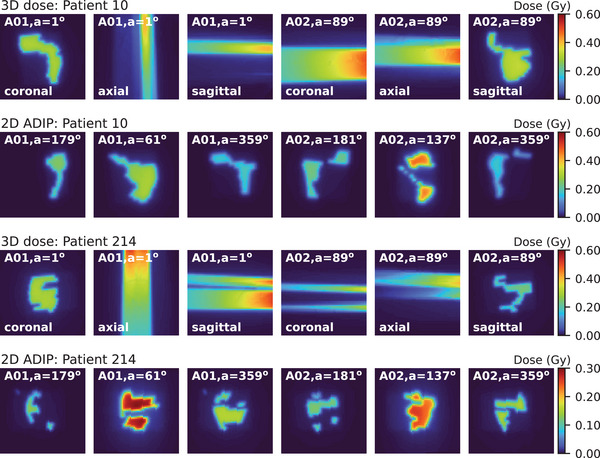
Illustrations of the input data for two patients. The three‐dimensional doses per control point (3D dose) are shown in coronal, axial, and sagittal planes for the arc (A) and gantry angle (a). The two‐dimensional average dose intensity projections (2D ADIP) are shown in the beam‐eye view angle.

**FIGURE 4:**
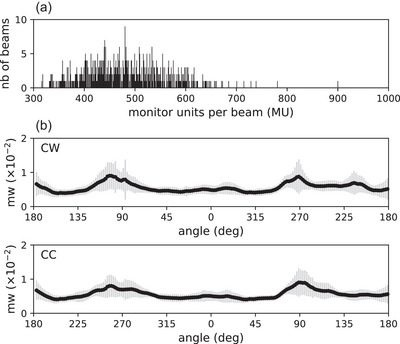
(a) Monitor units per beam frequency (MU) considered in this study (training, validation, and testing datasets). (b) Meterset weight (mw) per beam angle (deg) in the clockwise (CW) and counterclockwise (CC) arc.

A total of 302 plans were selected and split into 220/40/42 patients for training/validation/testing of the AI models. Illustrations of three‐dimensional dose distributions per control point and ADIPs used as input are shown in Figure 3 for different patients. The distribution over all patients of the clinical monitor units per beam and meterset weights per control point in clinical plans is shown in Figure 4. The monitor units per beam ranged between 316 and 901 MU (median = 479 MU, *n* = 302). In both clockwise and counterclockwise beams, the meterset weight standard deviation was maximized at gantry angles of 90^o ^± 10° and 270^o ^± 10°, corresponding to gantry angles in which the beam traverses femoral heads.

### 3.2 Model performance

**FIGURE 5:**
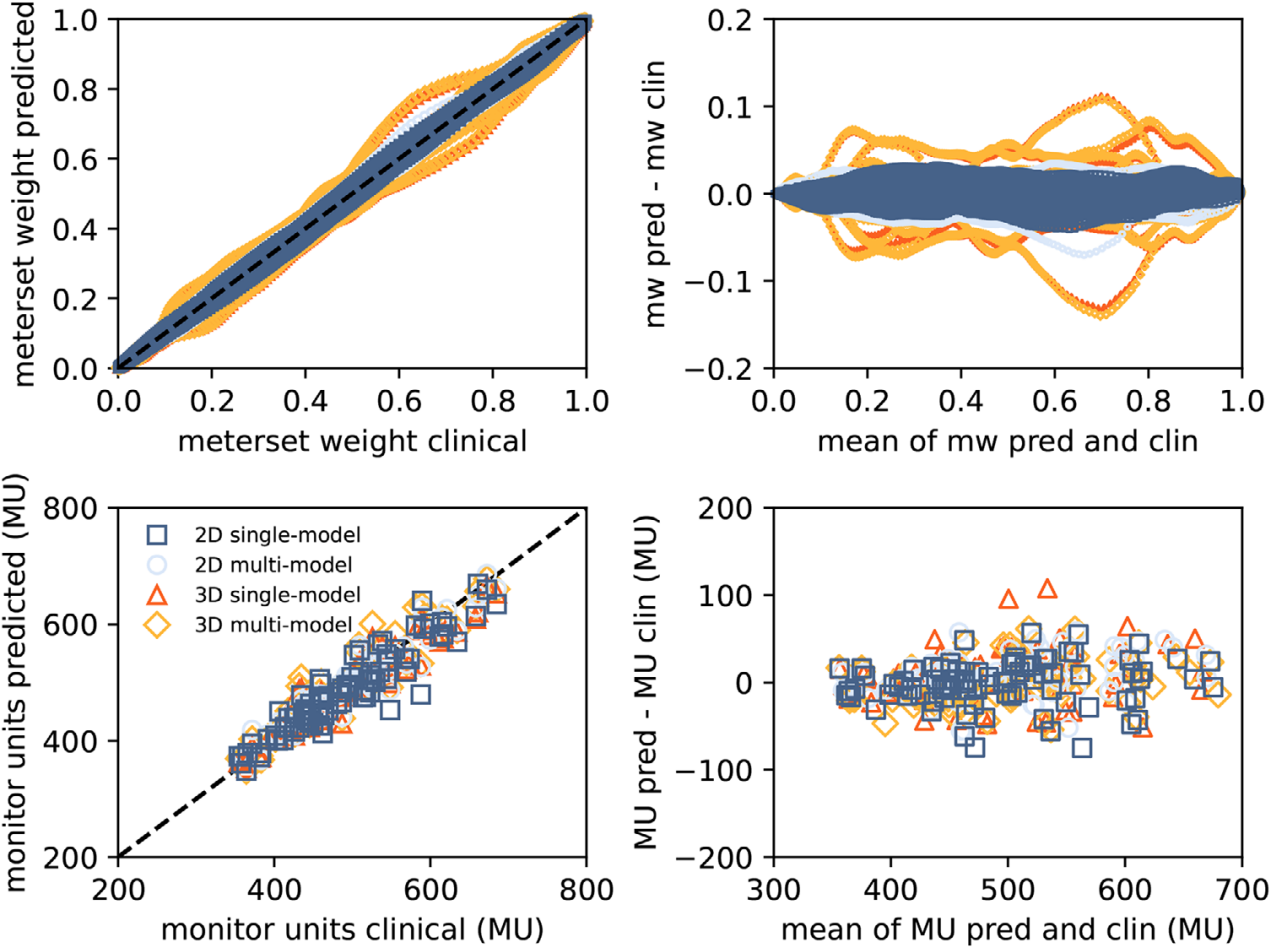
(Top) Predicted (pred) versus clinical (clin) meterset weight (mw) per control point (left) and its associated Bland–Altman plot (right). (Bottom) Predicted versus clinical monitor units per beam (MU) (left) and its associated Bland–Altman plot (right). The legend shown applies to all plots.

The times to create the AI‐RTPlan per fold per patient were 0.6/6.2 s for the 2D/3D single‐model and 1.7/ 2.1 min for the 2D/3D multi‐model. The differences in training and validation MAPE were lower than 2% across folds in all models. The predicted and clinical meterset weights for patients in the testing dataset are shown in Figure 5 for the four scenarios. The correlation between the predicted and clinical meterset weight was greater than 0.99 (*p*‐values < 0.05) in the four approaches. The mean errors with respect to the clinical plan were mean ± SD = ‐0.4 ± 3.8%/−0.2 ± 4.8% for the 2D/3D single‐model and 0.01 ± 3.9%/−0.1 ± 5.0% for the 2D/3D multi‐model. The predicted and clinical monitor units per beam are shown in Figure 5. The correlation between the predicted and clinical monitor units was greater than 0.93 in all approaches (*p*‐values < 0.05 in all cases). The mean errors with respect to the clinical monitor units were ‐0.9 ± 5.5%/−1.2 ± 4.5% for the 2D/3D single‐model and 0.4 ± 4.8%/0.5 ± 5.2% for the 2D/3D multi‐model.

### 3.3 Dose comparison

After importation of the AI‐RTPlans in the TPS and dose calculation, differences in the normalization factor applied to optimize clinical goal achievement were mean ± SD (min, max) = −0.1 ± 5.5% (−11.4%, 18.0%)/−0.4 ± 4.3% (−11.0%, 9.0%) in the 2D/3D single‐model, −2.1 ± 4.5 (−13.0%, 10.0%)/−2.2 ± 5.3 (−17.3%, 9.0%) in the 2D/3D multi‐model, and −5.4 ± 15.3% (−36.0%, 37.0%) in the average approach. The gamma passing rates were mean ± SD (min, max) = 99.4 ± 1.5% (93.3%, 100.0%)/96.9 ± 9.5% (59.7.8%, 100.0%) in the 2D / 3D single‐model, 99.9 ± 0.3% (98.3%, 100.0%)/96.7 ± 10.1% (57.9%, 100.0%) in the 2D/3D multi‐model, and 85.9 ± 8.8% (58.4%, 99.1%) in the average approach. In all cases, normalization improved gamma passing rates as compared to unnormalized plans (*p*‐values < 0.001 in all comparisons).

### 3.4 Model evaluation

**FIGURE 6:**
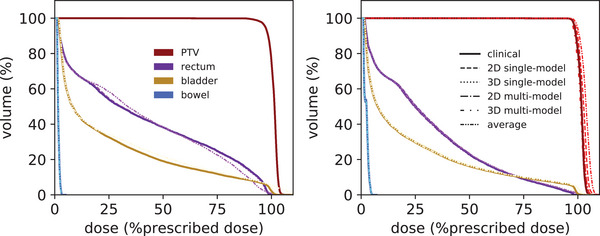
Dose‐volume histograms for one patient representative of the median normalization factor (left) and the patient with the lowest normalization factor (right). Results for the PTV, rectum, bladder, and bowel are shown obtained with the clinical value, AI predictions (2D / 3D single‐ and multi‐model), and the average of the monitor units over all patients.

**FIGURE 7:**
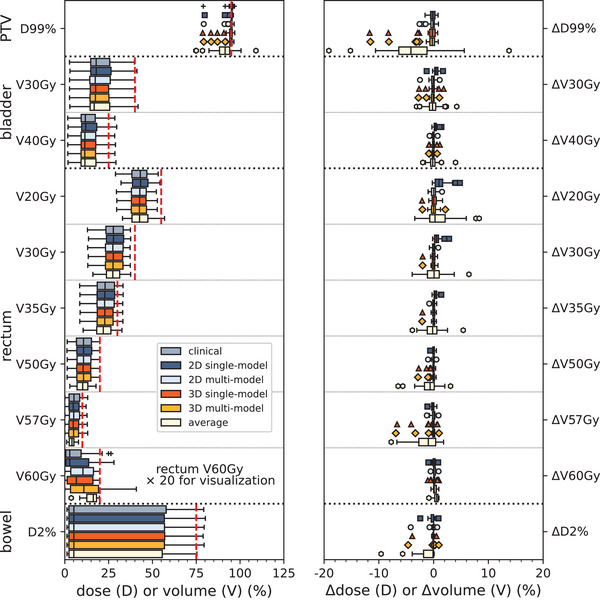
(Left) Boxplots of dose‐volume metrics (either dose (D) in % of the prescribed dose or volume (V) in % of the total volume) evaluated in this study. The red dashed line indicates the dose limit. Results of rectum V60Gy (dose limit = 1%) were multiplied by 20 for visualization. (Right) Dose (Δdose) or volume (Δvolume) metric difference (%) between AI models and clinical plans.

Dose‐volume histograms for a patient representative of the median normalization factor and the patient with the lowest normalization factor are shown in Figure 6. Boxplots of the clinical and predicted dose‐volume metrics are shown in Figure 7. In the clinical scenario, PTV coverage was compromised in 8 (19%) patients to spare OARs (PTV D99% ranged between 47.4 and 56.9 Gy in these patients). Dose limits were exceeded in bladder V30Gy [1 (2.4%) patient], rectum V35Gy [2 (4.8%) patients], rectum V57Gy [3 (7.1%) patients], rectum V60Gy [5 (11.9%) patients], and bowel D2% [1 (2.4%) patient]. The dose to femoral heads was negligible (< 0.01% of the prescription dose) in the clinical case and all approaches.

In the 2D single‐model and 2D multi‐model, target coverage was within ± 2% with respect to the clinical value in all patients of the testing dataset. In 39/42 (93%) patients, the same number or one extra clinical goal was achieved in AI‐RTPlans as compared with clinical plans in both models. In the three patients with less clinical goals, only one goal was not achieved in both 2D models. In the three patients with less clinical goals achieved in AI‐RTPlan in the 2D single‐model, all dose‐volume metrics were within ± 2% with respect to the clinical value. One of these patients had bladder V30Gy and V40Gy above the limits in the AI‐RTPlan, whilst only bladder V40Gy was above the dose limit in the clinical case. The two other patients had PTV D99% no more than 0.16% below the dose limit. Out of the three patients with less clinical goals achieved in AI‐RTPlan in the 2D multi‐model, PTV D99% was no more than 0.48% below the dose limit but PTV ΔD99% was within ± 2% with respect to the clinical value. All other dose‐volume metrics in AI‐RTPlans were within ± 2% with respect to the clinical value.

In the 3D single‐model and 3D multi‐model, 34 (81%) and 35 (83%) patients had achieved the same number or one more clinical goal in AI‐RTPlans as compared with clinical plans. The two approaches were characterized by a loss in PTV coverage; 5 (12%) and 2 (5%) patients had PTV ΔD99% lower than −2% with respect to the clinical value and PTV D99% lower than the clinical goal in the 3D single‐model and 3D multi‐model, respectively. Target underdosage resulted in low OAR dosage in both 3D models.

In the average approach, 22 (52%) patients had the same number of clinical goals achieved in AI‐RTPlans as compared to clinical plans. The average approach yielded the worst results, characterized by target underdosage and OAR overdosage.

## 4 DISCUSSION

A machine learning approach to predict machine parameters controlling the dose magnitude per control point in curative prostate cancer was introduced in this work. This is the first attempt to construct and study networks specialized in the prediction of the meterset weight per control point and monitor units per beam, two essential quantities for the development and integration of artificial intelligence in treatment planning. In this feasibility study, we demonstrated that convolutional neural networks can predict the dose magnitude per control point with satisfactory accuracy. The resulting treatment plans with predicted machine parameters had a similar quality to their clinical counterparts but were generated in a fraction of the time.

Our results are summarized in Table 2. The best predictions were obtained with the 2D single‐model and 2D multi‐model. In both cases, the dosimetry was comparable to the clinical case; the number of achieved clinical goals was higher or equal to the clinical case in more than 93% of patients in both models. However, 3D models did not provide acceptable dosimetry and were both characterized by a loss in target coverage.

**TABLE 2:** Summary of the results shown in this work.

		Single‐model		Multi‐model		
		2D	3D	2D	3D	MU average
Inference time per fold per patient	(s)	0.6	6.2	102	126	∅
Error in meterset weight per CP	(%)	−0.4 ± 3.8	−0.2 ± 4.8	0.01 ± 3.9	−0.1 ± 5.0	∅
Error in predicted monitor units per beam	(%)	−0.9 ± 5.5	−1.2 ± 4.5	0.4 ± 4.8	0.5 ± 5.2	∅
Gamma passing rates (3%/3mm)	(%)	99.4 ± 1.5	96.9 ± 9.5	99.9 ± 0.3	96.7 ± 10.1	85.9 ± 8.8
Achieved clinical goals	(%)	93	81	93	83	52

Abbreviation: CP, control point.

In terms of inference times, single‐models were faster than multi‐models, of the order of seconds versus minutes for a single patient. This difference was expected, as the model was loaded only once in the single‐model approache, and monitor units per control point were predicted in batch. However, in the multi‐model approach, each model had to be loaded per control point and monitor units had to be predicted as a sole sample. As a result, the use of a single GPU in the multi‐model approach severely impairs its clinical applicability. However, the inference time may be reduced by implementing distributed computing in the multi‐model approach and, with an appropriate number of GPUs, be equivalent to the single‐model approach.

Our results may be compared with an earlier investigation that predicted both MLC leaf positions and monitor units per beam to generate AI‐RTPlan for prostate cancer.^7^ The agreement between the calculated dose generated with predicted and clinical machine parameters as obtained with 2D models was improved in this study (minimum passing rate > 93% in both 2D models) as compared with the previous investigation (average gamma passing rate = 92%, minimum passing rate = 70%). Furthermore, the target coverage was within ± 2% of the clinical values in all patients in the 2D single‐model and 2D multi‐model of this study, while the PTV coverage was slightly compromised in the earlier study (PTV D98% = 92.9%/95.7% in predicted/clinical plans). Moreover, both studies led to comparable results for OARs, for which dose‐volume metrics were comparable between predicted and clinical plans. It is worth noting that the earlier study was more challenging, as the MLC leaf positions and the monitor units were predicted altogether. We rather propose to consider the prediction of the dose magnitude and shape independently from each other to improve the accuracy in both approaches before combining them. The methods developed in this study may therefore be integrated into future AI VMAT treatment planning workflows for accurate prediction of the monitor units per control point.

This study considered a homogeneous set of treatment plans in terms of anatomical site, prescription dose, fractionation, and number of control points. This choice was made to assess the feasibility of the method. It is expected that the models developed in this work will only apply under these specific conditions. Models capable of incorporating multiple sites, prescription doses, fractionations, and numbers of control points are yet to be investigated.^19^ Data augmentation was not used in both single‐models due to memory limitations, especially for the 3D model. Strategies involving distributed learning to allow data augmentation may have improved the results. The learning rates and early stopping criteria were empirically optimized to achieve convergence and limit computational resources. A future detailed investigation of the effect of these parameters on the model performance would be interesting. Control points are interdependent, and this work did not consider their relationship. Architecture based on time series prediction implemented with distributed computing may grasp this interdependence and additionally improve the results. Furthermore, only dose distributions were considered as input in this work. Information about patient attenuation and or organ segmentation in a multi‐channel strategy and or beam energy may have improved the results. It is worth noting that plan quality was evaluated with the number of achieved clinical goals and the difference between dose‐volume metrics in predicted and clinical plans. This evaluation remains subjective, as there are currently no accepted standards to evaluate plan quality.^20^ It is worth noting that the number of folds was constant in this work. An investigation of the robustness of the predictions through variations of the number of folds would be interesting.

The network assumed that three‐dimensional dose distributions per control point or their associated ADIPs were provided. In a real‐case scenario, these dose distributions will be predicted from either a clinical dose distribution (which would also be predicted) or a planning of CT and organ segmentations. In the context of AI VMAT treatment planning, any error in each building block (organ segmentation prediction, dose distribution prediction, multileaf collimator (MLC) leaf positions prediction, and monitor units per control point prediction) is expected to be propagated over the whole chain. Therefore, any attempt to improve the accuracy of each individual prediction is expected to be beneficial. To our knowledge, no studies have yet addressed the prediction of the three‐dimensional dose per control point. We intend to develop such an approach as the next step for the accurate prediction of machine parameters.

## 5 CONCLUSIONS

Accurate prediction of monitor units per control point from models trained on a homogeneous set of treatment plans for curative prostate cancer is feasible. The predicted treatment plans had a similar quality to their clinical counterpart but were generated in a few seconds. The methods developed in this study may be integrated into a greater workflow aiming at the prediction of radiation therapy treatment plans.

## AUTHOR CONTRIBUTIONS

Mathieu Gaudreault, Lachlan McIntosh, Nicholas Hardcastle, and Vanessa Panettieri conceived and designed the study. MG performed the data collection and conducted the analysis. Katrina Woodford, Jason Li, Susan Harden, and Sandro Porceddu supervised the project throughout its stages. All authors reviewed and approved the final version.

## CONFLICT OF INTEREST STATEMENT

Nicholas Hardcastle receives research funding from Varian Medical Systems for unrelated work. Nicholas Hardcastle is a paid consultant of SeeTreat Medical. Sandro Porceddu receives funding from Metro South Hospital for unrelated work. Sandro Porceddu is a paid consultant of the Steering Committee of cPOST.
